# Urinary Methylmalonic Acid as an Indicator of Early Vitamin B12 Deficiency and Its Role in Polyneuropathy in Type 2 Diabetes

**DOI:** 10.1155/2014/921616

**Published:** 2014-02-26

**Authors:** Ai-li Sun, Yi-hong Ni, Xiao-bo Li, Xiang-hua Zhuang, Yuan-tao Liu, Xin-hua Liu, Shi-hong Chen

**Affiliations:** ^1^The Second Hospital of Shandong University, 247 Beiyuan Street, Ji'nan, Shandong 250033, China; ^2^Qingdao Haici Hospital, Qingdao 266033, China; ^3^Department of Endocrinology, The Second Hospital of Shandong University, 247 Beiyuan Street, Ji'nan, Shandong 250033, China

## Abstract

The rising incidence of diabetes and its negative impact on quality of life highlights the urgent need to develop biomarkers of early nerve damage. Measurement of total vitamin B12 has some limitations. We want to determine the levels of urinary methylmalonic acid and its relationships with serum vitamin B12 and polyneuropathy. The 176 Chinese patients with Type 2 diabetes mellitus were divided into 3 groups according to the levels of vitamin B12. A gas chromatography mass spectrometric technique was used to determine blood methylmalonic acid and urinary methylmalonic acid. The diagnosis of distal diabetic polyneuropathy was based on the determination of bilateral limb sensory and motor nerve conduction velocity and amplitude with electromyogram. Multiple regression analysis revealed that urinary methylmalonic acid/creatinine, blood methylmalonic acid, and so forth were variables that influenced diabetic polyneuropathy significantly. Nerve sensory conduction velocity and nerve amplitude in the group of urinary methylmalonic acid/creatinine >3.5 mmol/mol decreased significantly. Superficial peroneal nerve sensory and motor conduction velocity and ulnar nerve compound motor active potential amplitude were inversely correlated with urinary methylmalonic acid/creatinine. Urinary methylmalonic acid correlates with serum vitamin B12 levels in person with diabetes and is a sensitive marker of early polyneuropathy.

## 1. Introduction

Diabetes mellitus (DM) is caused by genetic and environmental interactions, along with changing lifestyles and an aging population, increasing incidence of diabetes. Diabetic neuropathy is one of the major complications of diabetes with both Type 1 and Type 2. Up to 50% of all diabetes have polyneuropathy which is a major cause of morbidity and associated with increased mortality, and up to 26% of diabetics develop painful diabetic neuropathy with debilitating effects on quality of life [1–3].

The rising incidence of diabetes and its negative impact on quality of life highlights the urgent need to develop biomarkers of early nerve damage. The gold-standard method to evaluate morphological change in small nerve fibres was the skin biopsy [4]; however, this technique is limited by cost and invasiveness, provides no information about the function of nerve fibres, and cannot be employed as a generalized screening test in all patients.

Vitamin B12 (Vit B12) is a cofactor for methylmalonylCoA mutase, which converts methylmalonyl CoA to succinyl CoA. Measurement of total Vit B12 suffers from some limitations; in particular, most of the cobalamin measured is that bound to haptocorrin (HC) [5, 6]. Methylmalonic acid (MMA) may contribute to neuronal injury in many human conditions in which it accumulates, including methylmalonyl-CoA mutase and Vit B12 deficiency [7, 8]. The impaired activity of the enzyme leads to an accumulation of MMA and an elevated plasma concentration [9]. MMA is biochemically more stable in urine than in serum and has a 40-fold greater concentration in urine [10]. Urinary methylmalonic acid (uMMA) concentration offers a potentially useful functional marker of Vit B12 status. Moreover, the measurement of uMMA would be a less invasive method for the purpose of screening or epidemiologic studies. In addition, uMMA is excreted very efficiently by the kidneys, which concentrates the metabolite in the urine and makes it a sensitive indicator of tissue depletion [10].

Based on the above considerations, we believed that uMMA was an intermediate metabolite during the course of neuropathy, and there was an important relationship between uMMA and the formation of neuropathy. The aim of the present study was to assess uMMA levels in patients presenting with and without diabetic polyneuropathy (DPN) and to determine the role of uMMA in DPN.

## 2. Subjects, Materials, and Methods

### 2.1. Subject Selection

The study population consisted of 176 Chinese Han patients (male 97 and female 79) with Type 2 diabetes mellitus aged 38–70 y recruited upon admission to the Second Hospital of Shandong University between June in 2009 and June in 2011 who were eligible for the study.

Exclusion criteria were as follows: history of serious heart and lung disease, diabetic kidney disease or other kidney diseases, pernicious anemia, intestinal surgery, and gastrointestinal disease, excluding antibiotics, colchicine, aminosalicylic acid, H2 receptor antagonists and proton pump inhibitors and other effects of gastrointestinal motility drugs, and the patients with the Vit B12 or methylcobalamin treatment. Long-term vegetarians (more than six months) and those refused concurrent electrophysiological or laboratory testing were excluded too.

According to the level of Vit B12, the patients were divided into 3 groups: group A (Vit B12 < 180 ng/L, *n* = 58), group B (Vit B12 180–400 ng/L, *n* = 68), and group C (Vit B12 > 400 ng/L, *n* = 50).

### 2.2. Detection of Indicators

The fasting blood samples (22 mL/person) via venipuncture into EDTA-coated tubes (5 mL) for a full blood were counted to check glycosylated hemoglobin A1c (HbA1c), triglycerides (TG), high-density lipoprotein cholesterol (HDL), low-density lipoprotein cholesterol (LDL), serum creatinine (sCr), Vit B12, folic acid, ferritin, homocysteine (Hcy), hemoglobin (HGB), and mean corpuscular volume (MCV). Holotranscobalamin (holoTC) level was determined by microparticle enzyme immunoassay on Axsym analyzer (both from Abbott, USA). Fasting morning urine samples were for use. Serum samples were stored at −80°C. Approximately 10 mL fasting urine samples were stored at −80°C untreated.

The application of stable isotope dilution method for rapid extraction of the solid phase sample by gas chromatography mass spectrometry was used for the determination of blood methylmalonic acid (bMMA) and uMMA. Urinary MMA was expressed relative to urinary creatinine as the uMMA/creatinine.

Diabetic polyneuropathy: the diagnosis of bilateral limb sensory and motor nerve conduction velocity (SNCV/MNCV) and amplitude was performed, according to standard techniques protocols, by means of Keypoint, Dantec equipment, for all individuals. Tests were carried out in a warm room to minimize the effects of temperature on nerve conduction velocity (CV), and the limbs were warmed throughout with an infrared lamp to maintain a skin temperature over 35°C. Suprathreshold stimulation was used, the frequency was l Hz (feeling) and 1 Hz (movement), respectively, and course was both for 0.2 ms. Nerve conduction and electromyographic studies were performed in four limbs, which included 2 nerves of upper limbs and 2 nerves of lower limbs, by means of standard techniques. We measured the amplitude and velocity of motor nerve conduction and sensor nerve conduction in 4 nerves: superficial peroneal, sural, median, and ulnar.

### 2.3. Statistical Analysis

All experimental data were analysed by SPSS software 16.0 (SPSS, Chicago, IL, USA). Measurement data were presented as mean ± standard deviation (mean ± SD). Single factor and multifactor ANOVA analysis were for the mean difference among the groups. Correlation was analysed using Pearson correlation. The count data were analyzed by *χ* ^2^ test, and logistic regression analysis was used for an independent risk factor of diabetic polyneuropathy. *P* < 0.05 was considered statistically significant.

## 3. Results

### 3.1. Analysis of Clinical Characteristics (See Table 1)

Patients in the two groups with normal Vit B12 had decreased serum concentrations of HDL, ferritin, folic acid, holoTC, and HGB and increased uMMA/creatinine and bMMA compared to those lack Vit B12. In addition, DM Type 2 patients with Vit B12 over 400 ng/L had significantly increased ferritin, holoTC, and HGB and decreased uMMA/creatinine compared to those with Vit B12 180–400 ng/L. There was no significant difference in LDL, MCV, duration, sCr, TG, BMI, and HbA1c among the three groups. Polyneuropathy was present in 24.7% of our diabetic patients using electromyogram.

### 3.2. Multiple Regression Analysis

Multiple regression analysis revealed that uMMA/creatinine (*P* = 0.001), bMMA (*P* = 0.02), diabetes duration (*P* = 0.045), and holoTC (*P* = 0.003) were variables that influenced DPN significantly and independently while the serum concentration of Vit B12 did not have an independent significant influence on DPN (Table 2).

### 3.3. Prevalence of Electrophysiological Markers of Neuropathy Severity according to Urinary Methylmalonic Acid/Creatinine

To value the relationship between uMMA/creatinine and DPN, we separated data according to whether uMMA/creatinine was normal or elevated. It was observed that nerve sensory conduction velocity and nerve amplitude in the group of uMMA/creatinine >3.5 mmol/mol decreased significantly. All individual nerve conduction parameters except median nerve sensory and motor conduction velocity and ulnar nerve motor conduction velocity were statistically significant between the two groups (see Table 3).

### 3.4. The Correlation Analysis between Urinary Methylmalonic Acid/Creatinine and Electrophysiological Markers of Neuropathy Severity

Univariate analysis was carried out with the uMMA/creatinine as the outcome variable. Some significant correlations were revealed between the uMMA/creatinine and other electrophysiological markers of neuropathy severity (Table 4). Superficial peroneal nerve sensory and conduction velocity (*r* = −0.496, *P* = 0.0005; *r* = −0.327, *P* = 0.003, resp.) and ulnar nerve sensory active nerve potential amplitude (*r* = −0.315, *P* = 0.005) were inversely correlated with the uMMA/creatinine. Sural nerve compound motor active potential amplitude (*r* = −0.196, *P* = 0.02), sural nerve motor conduction velocity (*r* = −0.205, *P* = 0.01), superficial peroneal nerve compound motor active potential amplitude (*r* = −0.197, *P* = 0.015), and ulnar nerve sensory conduction velocity (*r* = −0.187, *P* = 0.02) showed modest inverse correlations with uMMA/creatinine.

## 4. Discussion

Our data showed polyneuropathy was present in 24.7% of our diabetic patients using electromyogram (EMG), which was higher than the previous report [11], maybe because of sample selection or the diagnostic criteria of neuropathy. We demonstrated a significant association between polyneuropathy and duration of diabetes in our patients and no association with age, while, in the Knuiman and Young studies, neuropathy was correlated with age and duration of diabetes [12, 13]. This may reflect the age distribution (all were below 70 years of age in the study).

The bMMA level in the patients with Vit B12 deficiency was significantly higher than in the people with normal Vit B12, while there was no significant difference between the two groups with normal Vit B12, which meant bMMA was not sensitive for early vitamin B12 deficiency. It is not considered the gold standard for the determination of Vit B12 deficiency [14], because its concentrations are also elevated in renal failure, thyroid disease, small-bowel bacterial overgrowth, and conditions of hemoconcentration [15].

In the study, holoTC gradually reduced accompanied with Vit B12 decrease. There was great difference among the three groups, and it was closely related to DPN. We thought that holoTC is a sensitive marker for Vit B12 deficiency, as previously reported [16]. HoloTC measurement has come of age; however, it has not acquired wide clinical acceptance to date, maybe due to the test's cost and limited availability [17].

The present results demonstrated that when part of Vit B12 was in normal range, uMMA had obviously exceeded the normal level, and there was great difference among the three groups, so we thought that uMMA was sensitive for the early deficiency of cobalamin, consistent with the previous reports [18, 19]. The fasting urinary MMA concentration corrected for creatinine was highly correlated with diabetic polyneuropathy. The mechanism was unclear, and the probable mechanism was that methylmalonic acid affected energy metabolism and the citric acid cycle and then led to neurotoxicity and the occurrence of DPN. In addition, uMMA was reported related to the enhanced inflammation in acute myocardial infarction [20]. The uMMA measurements are a noninvasive method that requires only a 1 mL urine specimen, and for months if frozen, it may offer a new method of analysis for identifying true tissue cobalamin deficiency [21].

The nerve conduction velocity and amplitude of four limbs decreased obviously as uMMA/creatinine was above normal. There was no significant difference between the decreased conduction velocity of median and ulnar nerves and the increased uMMA/creatinine. The upper limb sensory nerves appeared to be preferably affected by axonal damage as demonstrated by the finding that the sensory active nerve potential amplitude of the median and ulnar nerves was reduced in a remarkably high percentage of study patients, versus the conduction velocity of the two nerves [22]. The abnormality in the superficial peroneal and sural nerves in the lower limb was more severe than in the median and ulnar nerve. The presence of conduction block and abnormal amplitude in our patients also raises the possibility that it was a manifestation of a more widespread demyelinating polyneuropathy, according to [23]. The correlation analysis showed that the nerve conduction block of lower limbs was more serious in the group of the increasement of uMMA/creatinine.

Our findings were presented with some limitations. Although we identified patients prospectively, they were not randomly selected from a population with Type 2 diabetes with or without DPN. We underwent EMG for each patient. Being an invasive operation, EMG could not reflect early neuropathy screening, so some early neuropathy patients might be missed. We excluded the patients with Type 1 diabetes because of their expected metformin restriction and the distinct pathophysiological mechanisms, and further study would be conducted in depth.

In conclusion, uMMA and holoTC are significantly associated with cobalamin deficiency and polyneuropathy. The relationships among cobalamin deficiency, decline holoTC, elevated uMMA levels, and the polyneuropathy are controversial, and further work is needed to prove a direct causal relationship and the mechanism, but both cobalamin deficiency and elevation of its serum metabolites are associated with the presence of a sensorimotor peripheral neuropathy [24]. uMMA may exacerbate polyneuropathy as a result of other unknown mechanisms; a clear understanding of its role necessarily awaits further research on the pathogenesis of DPN. Despite these limitations, we believe that uMMA is a potential iatrogenic contributor to the severity of the polyneuropathy in the population described. It can be recommended that uMMA, alone and in combination with holoTC, has better diagnostic efficiency in diagnosing Vit B12 deficiency for the diabetic patients and is expected to become more sensitive predicting and monitoring indicators of diabetic polyneuropathy.

## Figures and Tables

**Table 1:** Comparison of the clinical data among the three groups.

Parameters	Group A	Group B	Group C
Age (year)	58.24 ± 10.53	55.81 ± 10.83	56.98 ± 10.72
Male/female	30/28	36/32	28/22
Duration (year)	6.28 ± 6.6	4.67 ± 5.88	4.13 ± 4.77
Vitamin B12 (ng/L)	127.53 ± 60.71	303.62 ± 109.98	662.86 ± 260.92
BMI (Kg/m^2^)	26.06 ± 3.61	26.10 ± 3.31	25.93 ± 4.09
Hemoglobin A1c (%)	9.12 ± 2.33	9.42 ± 2.13	8.38 ± 1.97
Triglycerides (mmol/L)	1.79 ± 0.86	2.12 ± 1.81	1.81 ± 0.88
High-density lipoprotein cholesterol (mmol/L)	1.20 ± 0.24	1.31 ± 0.28*	1.39 ± 0.31*
Low-density lipoprotein cholesterol (mmol/L)	2.72 ± 0.80	2.84 ± 0.63	2.01 ± 1.23
Serum creatinine (umol/L)	60.00 ± 4.32	65.89 ± 4.71	61.08 ± 4.46
Hemoglobin (g/L)	130.10 ± 18.54	136.65 ± 14.58*	146.71 ± 10.01^∗#^
Mean corpuscular volume (fL)	85.32 ± 5.60	86.66 ± 8.36	87.38 ± 9.98
Ferritin (ug/L)	166.44 ± 92.51	225.18 ± 103.06*	259.33 ± 119.33^∗#^
Folic acid (ng/L)	6.17 ± 2.06	6.50 ± 2.18*	6.58 ± 2.32*
Holotranscobalamin (pmol/L)	54.82 ± 30.47	70.10 ± 26.00^a^	81.32 ± 32.91^ab^
Urinary methylmalonic acid/creatinine	8.04 ± 2.19	4.15 ± 1.06^a^	2.75 ± 0.71^ab^
Blood methylmalonic acid (nmol/L)	400.09 ± 80.52	370.57 ± 110.9^a^	350.72 ± 120.02^a^
Metformin drug usage (%)	80	76	79
Polyneuropathy (%)	35	21	18

Values are means ± SD. *P < 0.05 compared to group A; ^#^ *P* < 0.05 compared to group B; ^a^ *P* < 0.01 compared to group A; ^b^ *P* < 0.01 compared to group B.

**Table 2:** Logistic analysis of influencing factors of diabetic polyneuropathy.

Parameters	OR value (95% CI)	*P* value
Age (year)	0.73 (0.84–1.08)	0.59
Duration (year)	1.543 (1.302–1.829)	0.045
Low-density lipoprotein cholesterol (mmol/L)	0.76 (0.63–0.89)	0.587
High-density lipoprotein cholesterol (mmol/L)	1.03 (0.81–1.42)	0.407
Triglycerides (mmol/L)	0.27 (0.11–0.36)	0.816
Serum creatinine (umol/L)	1.15 (0.79–1.54)	0.313
HbA1c (%)	1.19 (0.85–1.43)	0.254
Mean corpuscular volume (fL)	0.18 (0.11–0.25)	0.884
Hemoglobin (g/L)	0.99 (0.70–1.27)	0.458
Ferritin (ug/L)	1.08 (0.93–1.21)	0.340
Folic acid (ng/L)	0.92 (0.86–0.96)	0.532
Urinary methylmalonic acid/creatinine (mmol/mol)	4.07 (3.15–5.46)	0.001
Blood methylmalonic acid (pg/mL)	2.152 (1.799–2.42)	0.02
Vitamin B12	0.88 (0.794–0.997)	0.547
Holotranscobalamin (pmol/L)	3.89 (2.77–4.56)	0.003

**Table 3:** Prevalence of electrophysiological markers of neuropathy severity according to urinary methylmalonic acid/creatinine.

Electrophysiological markers of neuropathy severity	uMMAr > 3.5 mmol/mol	uMMAr < 3.5 mmol/mol	*P *
Sural nerve SNAP amplitude (*μ*V)	1.45 ± 0.41	2.31 ± 1.02	0.03
Sural nerve sensory conduction velocity (m/s)	45.35 ± 6.11	55.98 ± 9.25	0.04
Sural nerve CMAP amplitude (*μ*V)	1.12 ± 0.29	2.09 ± 0.91	0.001
Sural nerve motor conduction velocity (m/s)	40.06 ± 4.21	53.37 ± 7.24	0.005
Superficial peroneal nerve SNAP amplitude (*μ*V)	3.01 ± 1.07	5.56 ± 2.73	0.005
Superficial peroneal nerve sensory conduction velocity (m/s)	40.11 ± 3.03	47.32 ± 5.93	0.023
Superficial peroneal nerve CMAP amplitude (*μ*V)	5.03 ± 2.15	6.01 ± 3.39	0.012
Superficial peroneal nerve motor conduction velocity (m/s)	44.73 ± 4.91	54.12 ± 6.09	0.01
Median nerve sensory conduction velocity (m/s)	53.77 ± 5.16	55.36 ± 5.95	0.08
Median nerve SNAP amplitude (*μ*V)	9.25 ± 3.88	11.28 ± 5.06	0.034
Median nerve motor conduction velocity (m/s)	52.62 ± 4.41	56.15 ± 5.96	0.06
Median nerve CMAP amplitude (*μ*V)	7.31 ± 2.08	9.06 ± 3.16	0.02
Ulnar nerve sensory conduction velocity (m/s)	51.07 ± 5.49	58.10 ± 6.44	0.032
Ulnar nerve SNAP amplitude (*μ*V)	6.93 ± 2.01	8.36 ± 3.17	0.001
Ulnar nerve motor conduction velocity (m/s)	57.90 ± 6.25	61.32 ± 8.04	0.07
Ulnar nerve SNAP amplitude (*μ*V)	6.78 ± 1.84	8.16 ± 2.48	0.022

Key: SNAP: sensory active nerve potential; CMAP: compound motor active potential; uMMAr: urinary methylmalonic acid/creatinine.

**Table 4:** The correlation analysis between urinary methylmalonic acid/creatinine and electrophysiological markers of neuropathy severity.

Electrophysiological markers of neuropathy severity	*r *	*P *
Sural nerve SNAP amplitude (*μ*V)	−0.131	−0.06
Sural nerve sensory conduction velocity (m/s)	−0.120	−0.065
Sural nerve CMAP amplitude (*μ*V)	−0.196	−0.02
Sural nerve motor conduction velocity (m/s)	−0.205	−0.01
Superficial peroneal nerve SNAP amplitude (*μ*V)	−0.0034	−0.69
Superficial peroneal nerve sensory conduction velocity (m/s)	−0.496	−0.0005
Superficial peroneal nerve CMAP amplitude (*μ*V)	−0.197	−0.015
Superficial peroneal nerve motor conduction velocity (m/s)	−0.327	−0.003
Median nerve sensory conduction velocity (m/s)	−0.115	−0.07
Median nerve SNAP amplitude (*μ*V)	−0.089	−0.15
Median nerve motor conduction velocity (m/s)	−0.139	−0.055
Median nerve CMAP amplitude (*μ*V)	−0.002	−0.86
Ulnar nerve sensory conduction velocity (m/s)	−0.187	−0.02
Ulnar nerve CMAP amplitude (*μ*V)	−0.315	−0.005
Ulnar nerve motor conduction velocity (m/s)	−0.009	−0.60
Ulnar nerve SNAP amplitude (*μ*V)	−0.125	−0.065

SNAP: sensory active nerve potential; CMAP: compound motor active potential.

## References

[1] Davies M, Brophy S, Williams R, Taylor A (2006). The prevalence, severity, and impact of painful diabetic peripheral neuropathy in type 2 diabetes. *Diabetes Care*.

[2] Benbow SJ, Wallymahmed ME, Macfarlane IA (1998). Diabetic peripheral neuropathy and quality of life. *QJM*.

[3] Tesfaye S (2009). Advances in the management of diabetic peripheral neuropathy. *Current Opinion in Supportive and Palliative Care*.

[4] England JD, Gronseth GS, Franklin G (2009). Practice Parameter: evaluation of distal symmetric polyneuropathy: role of autonomic testing, nerve biopsy,and skin biopsy (an evidence-based review). Report of the American Academy of Neurology, American Association of Neuromuscular and Electrodiagnostic Medicine, and American Academy of Physical Medicine and Rehabilitation. *Neurology*.

[5] Lindenbaum J, Savage DG, Stabler SP, Allen RH (1990). Diagnosis of cobalamin deficiency: II. Relative sensitivities of serum cobalamin, methylmalonic acid, and total homocysteine concentrations. *American Journal of Hematology*.

[6] Matchar DB, McCrory DC, Millington DS, Feussner JR (1994). Performance of the serum cobalamin assay for diagnosis of cobalamin deficiency. *American Journal of the Medical Sciences*.

[7] Behrens MI, Koh J, Canzoniero LMT, Sensi SL, Csernansky CA, Choi DW (1995). 3-Nitropropionic acid induces apoptosis in cultured striatal and cortical neurons. *NeuroReport*.

[8] Zeevalk GD, Derr-Yellin E, Nicklas WJ (1995). Relative vulnerability of dopamine and GABA neurons in mesencephalic culture to inhibition of succinate dehydrogenase by malonate and 3-nitropropionic acid and protection by NMDA receptor blockade. *Journal of Pharmacology and Experimental Therapeutics*.

[9] Cox EV, White AM (1962). Methylmalonic acid excretion: an index of vitamin-B12 deficiency. *The Lancet*.

[10] Matchar DB, Feussner JR, Millington DS (1987). Isotope-dilution assay for urinary methylmalonic acid in the diagnosis of vitamin B12 deficiency. A prospective clinical evaluation. *Annals of Internal Medicine*.

[11] Lu B, Hu J, Wen J (2013). Determination of peripheral neuropathy prevalence and associated factors in Chinese subjects with diabetes and pre-Diabetes- ShangHai Diabetic neuRopathy epidemiology and molecular genetics study (SH-DREAMS). *PLoS One*.

[12] Knuiman MW, Welborn TA, McCann VJ (1986). Prevalence of diabetic complications in relation to risk factors. *Diabetes*.

[13] Young MJ, Boulton AJM, Macleod AF, Williams DRR, Sonksen PH (1993). A multicentre study of the prevalence of diabetic peripheral neuropathy in the United Kingdom hospital clinic population. *Diabetologia*.

[14] Clarke R, Refsum H, Birks J (2003). Screening for vitamin B-12 and folate deficiency in older persons. *American Journal of Clinical Nutrition*.

[15] Bjørke Monsen AL, Ueland PM (2003). Homocysteine and methylmalonic acid in diagnosis and risk assessment from infancy to adolescence. *American Journal of Clinical Nutrition*.

[16] Lindgren A, Kilander A, Bagge E, Nexø E (1999). Holotranscobalamin—a sensitive marker of cobalamin malabsorption. *European Journal of Clinical Investigation*.

[17] Nexo E, Hoffmann-Lücke E (2011). Holotranscobalamin, a marker of vitamin B-12 status: analytical aspects and clinical utility. *American Journal of Clinical Nutrition*.

[18] Flatley JE, Garner CM, Al-Turki M (2012). Determinants of urinary methylmalonic acid concentration in an elderly population in the United Kingdom. *American Journal of Clinical Nutrition*.

[19] Norman EJ (2004). Urinary methylmalonic acid test may have greater value than the total homocysteine assay for screening elderly individuals for cobalamin deficiency. *Clinical Chemistry*.

[20] Celik T, Kardesoglu E, Iyisoy A, Ozcan O, Kilic S, Yaman H (2009). Urinary methylmalonic acid in patients with acute myocardial infarction. *Medical Principles and Practice*.

[21] Norman EJ, Bernard MA, Firdaus M, Nakonezny PA, Kashner TM (1999). Urinary methylmalonic acid/creatinine ratio: a gold standard test for tissue vitamin B12 deficiency. *Journal of the American Geriatrics Society*.

[22] Rota E, Quadri R, Fanti E (2005). Electrophysiological findings of peripheral neuropathy in newly diagnosed type II diabetes mellitus. *Journal of the Peripheral Nervous System*.

[23] Acosta JA, Hoffman SN, Raynor EM, Nardin RA, Rutkove SB (2003). Ulnar neuropathy in the forearm: a possible complication of diabetes mellitus. *Muscle and Nerve*.

[24] Saperstein DS, Wolfe GI, Gronseth GS (2003). Challenges in the identification of cobalamin-deficiency polyneuropathy. *Archives of Neurology*.

